# How to Build Healthy Societies: A Thematic Analysis of Relevant Conceptual Frameworks

**DOI:** 10.34172/ijhpm.2023.7451

**Published:** 2023-11-07

**Authors:** Devaki Nambiar, Amy Bestman, Siddharth Srivastava, Robert Marten, Sonam Yangchen, Kent Buse

**Affiliations:** ^1^The George Institute for Global Health, New Delhi, India.; ^2^Faculty of Medicine, University of New South Wales, Sydney, NSW, Australia.; ^3^Prasanna School of Public Health, Manipal Academy of Higher Education, Manipal, India.; ^4^The Alliance for Health Policy and Systems Research, World Health Organization (WHO), Geneva, Switzerland.; ^5^The George Institute for Global Health, Imperial College London, London, UK.

**Keywords:** Healthy Societies, Health Governance, Health Policy, Governance, Social Determinants of Health, Structural Determinants of Health

## Abstract

**Background:** As the Sustainable Development Goals deadline of 2030 draws near, greater attention is being given to health beyond the health sector, in other words, to the creation of healthy societies. However, action and reform in this area has not kept pace, in part due to a focus on narrower interventions and the lack of upstream action on health inequity. With an aim to guide action and political engagement for reform, we conducted a thematic analysis of concepts seeking to arrive at healthy societies.

**Methods:** This paper drew on a qualitative thematic analysis of a purposive sample of 68 documents including political declarations, reports, peer reviewed literature and guidance published since 1974. Three independent reviewers extracted data to identify, discuss and critique public policy levers and ‘enablers’ of healthy societies, the "how."

**Results:** The first lever concerned regulatory and fiscal measures. The second was intersectoral action. The final lever a shift in the global consensus around what signifies societal transformation and outcomes. The three enablers covered political leadership and accountability, popular mobilization and the generation and use of knowledge.

**Conclusion:** Documents focused largely on technical rather than political solutions. Even as the importance of political leadership was recognized, analysis of power was limited. Rights-based approaches were generally neglected as was assessing what worked or did not work to pull the levers or invest in the enablers. Frameworks typically failed to acknowledge or challenge prevailing ideologies, and did not seek to identify ways to hold or governments or corporations accountable for failures. Finally, ideas and approaches seem to recur again over the decades, without adding further nuance or analysis. This suggests a need for more upstream, critical and radical approaches to achieve healthy societies.

## Background

 The COVID-19 pandemic, which pushed millions of people into extreme poverty, is described by the United Nations Development Programme (UNDP) as “the worst setback in a generation.”^1^ The pandemic has renewed recognition of the need to better address the drivers protecting and producing health.^2^ The United Nations (UN) has called for “ambitious plans that reimagine and rebuild health, social and economic systems.”^3^ This is echoed by the World Health Organization (WHO) in its calls for “a healthy recovery from COVID-19.”^2^

 The question however, is whether we want a recovery returning to our previous unhealthy and unsustainable state? Arguably, the world was primed for the pandemic and its inequitable impacts precisely because of a lack of meaningful action on the systemic and institutional drivers of health inequities. Until recently, improvements in economic progress have been accompanied with widening inequality, increased migration, growing urbanisation, decreased social mobility, expanding labour vulnerabilities, fraying social safety nets — in short, social conditions are worsening.^4,5^ The idea that we can “treat our way out” of the existing situation is increasingly untenable.

 Experiences within and beyond the health system provide insights into the kind of recovery possible – including what has been termed “healthy societies.”^6^ The idea of healthy societies builds on the Declaration of Alma Ata,^7^ the Ottawa Charter,^8^ and the WHO Commission on the Social Determinants of Health.^9^ It has also been advanced by other initiatives.^10^ While general statements are made and whole-of-society models have been endorsed in select geographies, there is limited clarity or agreement on the needed action and research agendas.

 We explore these themes and ideas as part of a larger narrative synthesis of the healthy societies literature reported in a companion paper.^6^ As mentioned in that paper, our aim was *to understand how several linked concepts inform healthy societies approaches, with the intention to inform political engagement for action, eventual policy interventions, and research in support of both. *Our analysis did not seek to develop a unifying theory, but focus on both the “what” and the “how” of healthy societies. In this second paper, we focus on the policy actions and research priorities (the “how”).

 Based on our prior work, we began with the premise that societal efforts for health are driven by policy levers, broadly defined as instruments used by governments to elicit system-wide and societal change to meet objectives and/or respond to key stakeholders.^11^ Governments can use and adjust policy levers to achieve such change.^11-13^ In the health sector, a policy lever can represent a discrete area of the system’s function.^12^ Levers operate across broader contexts; their implementation and effectiveness are conditioned by the presence of what are termed “enablers.” We defined enablers as broader conditions to be established or created alongside levers.

## Methods

 This study was initiated following a series of meetings and conversations that senior authors were involved with on defining the scope of healthy societies. These meetings and interactions relate to articulating what the WHO’s third Triple Billion target would encompass – it states that 1 billion more people should enjoy better health and well-being.^14^ Alongside this, some co-authors were involved with an institutional strategy-building on societal determinants of health and on articulating a research vision for “healthier societies.”

 These discussions and considerations were the starting point of our document selection which itself issued from two reviews, Maani et al^15^ (a review of how commercial determinants of health are represented in social determinants frameworks) and van Olmen et al^16^ (a review of health systems frameworks). Using these reviews as a base, we determined inclusion criteria and developed a sample of documents in the English literature starting from the 1974 Lalonde Commission Report.^17^ Our database included political declarations, commission and UN reports, peer-reviewed papers, commissioned academic evidence reviews and non-government organisation^8^ guidance notes. Additional documents were identified through Google Scholar searches, snowball sampling and author suggestions based on meetings and emerging literature in the course of the analysis, that at least two co-authors also determined to be relevant to defining and understanding the concept of ‘healthy societies.’^[1]^ (See Supplementary file 1).

 Ultimately, 68 documents were used as the database for extraction (See Supplementary file 2). Given the wide variation of type of documents and the aim to examine the ‘how,’ this analysis did not include a quality appraisal of texts used. Rather, we sought to explore the operationalization of frameworks, seeking to build the “how” from the “what” extracted in an earlier paper.^6^

 Three researchers independently extracted data from the included documents into a coding template. The initial analysis focused on basic details (date published, authors, affiliations, type of document, funder) and analytical information (aim, broad topics, policy approaches, recommendations, and action and research agendas advocated). Coding was discussed and revised and the framework revised and reapplied. Additional documents used to contextualise the findings were identified through citation chaining and iterative examination.

 Following this initial coding and indexing, a thematic, inductive approach to analysis was used, and the findings focused on key themes and implications, rather than the number of documents that refer to specific themes. The framework presented here, on levers and enablers, was developed iteratively through a focused extraction of ‘action agendas.’ These were defined as calls or recommendations made in the documents to any range of stakeholders to raise the salience of, take steps toward (including investments) or place renewed emphasis on an issue, value, way of doing things, and/or an idea. In addition to extracting text on specific policy levers and agendas, researchers noted text pertaining to accountability, and processes that would have to be in place for levers to function. These are categorized as “enablers.” Through several iterative drafts and discussions, the analysis identified that levers and enablers operate across multiple levels and interact. These have been indexed in relation to a ‘cube’ and finally presented in a narrative structure.

## Results

 This analysis found that levers and enablers play a range of critical roles to engender healthy societies through their interactions at local, national and global levels (See Figure). All sides of the cube are interlinked, in the way Gaventa’s power cube also conceives of the interlinkages of various dimensions of power.^18^ Our conceptualisation departs somewhat from Gaventa’s exegesis, however, in that the “power cube” specifies forms and spaces, while this “action cube” emphasizes levers and enablers. In presenting the results, policy levers are outlined first and then the enablers. We also mention challenges and barriers to enacting the enablers and pulling the policy levers as identified in the literature. There is no hierarchy implied in the order of presentation.

**Figure:**
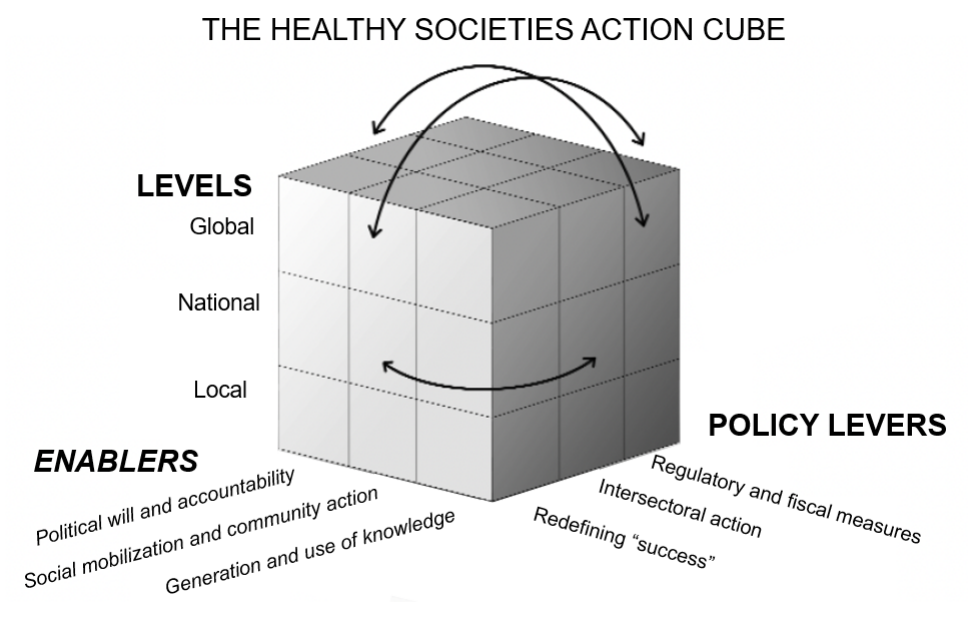
The Healthy Societies Action Cube, Adapted From the Power Cube by Gaventa.^18^

###  Levers

####  Lever 1 – Regulatory and Fiscal Measures

 Regulatory measures enforced by the state are described as key “mechanisms for change.” For example, Dahlgren and Whitehead argue that robust governmental social protection programmes are needed to address social and health inequities.^19^ Regulatory measures include reducing income inequality, promoting full and fair employment, providing social protection, and supporting equitable health financing.^3,9,19-23^ Many documents focus on governmental regulation, while others focus on hybrid models as well as the drawbacks of industry self-regulation. Legislation and other expressions of state-led regulation are critical to the four components of healthy societies described in our first paper (People, Places, Products, and Planet). For example, the predominant domestic regulatory measures discussed in the literature were taxes on tobacco and alcohol products and marketing regulations, as well as regulation of marketing aimed at children, including digital media, where it was suggested that government efforts to protect health are at present insufficient.^24,25^ Some argue that for non-communicable disease (NCD) prevention, inadequate state intervention, self-regulation by industry and weak accountability mechanisms all limit action on upstream drivers.^26^

 Many documents call for supportive trans- or inter-national regulation through regional and global agreements and treaties (such as in the trade sector),^27,28^ that reduce inequalities between and across nations.^29^ Some call for examination of new and binding framework conventions similar to the WHO Framework Convention on Tobacco Control.^26,30^ This includes, for example, global agreements on childhood obesity, on alcohol and health-harming products/industries^30^ and on food systems.^31^ A number of the documents draw attention to the need to protect regulatory processes from undue industry interference.^31-33^ Others argue that the existing human rights machinery must be used to support and hold countries (and corporations) accountable to established standards in existing agreements.^26^

 Documents also refer to industry self-regulation and hybrid forms of public-private regulation. Many analysts are concerned that the profit motive tends to make these approaches to regulation less effective that state-led regulation.^26^ Weaknesses of self-regulation include shallow commitments (or low standards), inadequate monitoring mechanisms (including those that lack independence), and a lack of enforcement. Where hybrid and self-regulation prevail, there is a need for “robust and independent accountability mechanisms” and capacity building to “negotiate with the private sector” on government-determined and -led activities.^30^ Regulation requires a combination of long-term and dynamic capabilities in the public sector.^34^

 The frameworks and literature we found support an interventionist and redistributive state.^3,9,17,19,22,23^ Calls relating to fiscal measures include investing more in interventions that control the determinants of health.^9,24,30,31,35,36^ There are also exhortations to remove health harming subsidies,^1,27^ including those propping up and/or incentivising the fossil fuel industry,^31^ or agricultural ones that prevent nature-based solutions and trap farmers on degraded lands. Fiscal incentives may increase the efficiency of carbon pricing and help channel private sector investment into long-lived, low-carbon technologies.^1^ Some documents called for “double duty” and “triple duty actions.”^31^ For example, in response to COVID-19, some economic stimulus programmes supported transitions to greener sustainable economies, leading to greater equity, resilience and sustainability.^3^ Stimulus programs are described as double duty benefits in that they reduce NCDs and protect the planet.^30^

 There are calls for greater research on more impactful and equitable fiscal measures. This includes assessing cross-sectoral policies, new technologies, and products. There is a particular focus in many research agendas on health co-benefits (and co-harms), including the distribution of co-impacts and their equity considerations.^27^ Research themes identified include how best to: (*i*) reduce and repurpose harmful subsidies; (*ii*) develop and implement appropriate taxes that promote sustainability, improve health, and reduce inequities; (*iii*) support local sustainable development initiatives that foster health; and (*iv*) regulate harmful activities.^27^

####  Lever 2 – Intersectoral Action

 The need for purposeful and coordinated engagement of sectors beyond the health ministry or department of health features prominently in early documents^8,17^ and is reiterated in subsequent frameworks.^23,37-40^ This is referred to as “intersectoral action” in a number of documents, defined as “a recognized relationship between part or parts of the health sector and part or parts of another sector, that has been formed to take action on an issue or to achieve health outcomes (or intermediate health outcomes) in a way which is more effective, efficient or sustainable than could be achieved by the health sector working alone.”^41^ Variants of such initiatives include multi- and trans-sectoral action (and policy and governance). In 1978, the Alma-Ata Declaration called for “national health policies and plans [to] take full account of the inputs of other sectors bearing on health.”^7^ There are also calls for greater integration and policy coherence between policy efforts.^40^ Among the most prominent frameworks is that of “healthy public policy” – which is achieved through a Health-in-All-Policies (HiAP) approach.^23^ This literature includes calls for strengthened legal obligations to embed health into policy making across sectors.^23,42^ The WHO European Regional office has published a number of strategies to enhance governance to facilitate multisectoral action.^19,24^ These and other documents advocate for more widespread use of health impact assessments of relevant policies across all ministries.

 Yet efforts to realize inter- or multi-sectoral action have had limited impact as health actors have struggled to link with other sectors and develop compelling messaging which resonates beyond public health and academic communities.^43^ These challenges reflect the complexity and challenges of intersectoral work.^44^ For example, a summary of intersectoral action in the field of women, child and adolescent health found limited evidence on the effectiveness of the health sector,^45^ with some calls for indicators to measure collaborative relationships.^19,24^

 The literature considers whether health or other sectors ought to lead on intersectoral action for health – and most of the literature suggests that the health sector holds this obligation. At the global level, some documents called for multilateral organizations (such as WHO) to support countries to coordinate HiAPs approaches across other sectors. Many of the documents consider various challenges of integrating health concerns into public policy beyond the health sector. In a more recent paper, a typology of roles of the health sector vis-à-vis other actors has been proposed; the health sector could lead, be in bi- or tri-lateral collaboration, support or otherwise have a minimal role. More recent papers explore the role of theory in informing multi-sectoral action and the mechanisms that enable effective intersectoral policy-making. These recent contributions advance the field and aim to pivot conversations from descriptive to strategic analysis. This includes evidence on the “political economy” of intersectoral action, including how ideas, institutions and interests influence intersectoral action outcomes (particularly in low- or middle-income country [LMIC] contexts) and the need for distinction between inter-, multi-, and trans-sectoral action, policy and governance.^46^

####  Lever 3 - Redefining Measures of Progress

 The final lever reflects calls for a recalibration of definitions of societal progress–reconsidering ultimate goals in public policy making. Several frameworks question the reliance on gross domestic product or gross national income type measures, and instead propose new approaches for societal well-being and sustainable development, like the Human Development Index. Some argue that COVID-19 provided an opportunity for such recalibration, and that rather than returning to “normal” we should ask what sort of societies we want. This includes calibrating progress in terms of how solutions address the challenges faced by people experiencing marginalisation, and the taking a longer-term view of impacts on and relationship of people and planet.

 Suggestions for redefining progress include placing greater emphasis on human welfare values (and indicators) that prioritise justice, inclusion and transparency.^17,27,47-49^ An obvious approach is to focus on equity metrics rather than metrics that look at averages or only outcomes in the poor.^19^ Multiple authors propose research on and integration of equity indicators and/or disaggregation of data and targets.^21,50-53^ Part of the process of establishing alternative goals and metrics involves acknowledging the relationship between health and economic well-being and the role of “institutionalised prejudices and administrative inefficiencies.”^21^

 Calls are made for indicators that move beyond measures of illness to measures of well-being. One suggestion is to assess all public policy against their impact on life satisfaction, availability of social support, percentage of the population with improved sanitation facilities, income distribution, unemployment rates and the proportion of primary school aged children not enrolled.^54^ However, some noted that well-being is not a clearly defined concept, calling for more research on indicators based on eudaimonic areas (realising one’s fullest potential) as metrics of progress and success. Similarly, others call for focusing research on mental well-being. Here, too, health must be seen in broader context: well-being is determined by our ability to obtain quality education, food and housing, among other factors.^55^

 A few models and ways to measure well-being have been advanced,^56-59^ including that by the Wellbeing Economy Alliance.^60^ Bhutan’s The Gross National Happiness Index (GNHI) suggests centering a population’s happiness and well-being with four principles: sustainable and equitable economic development; conservation of the environment (related to people’s relationship with a healthy and sustainable natural environment); preservation and promotion of culture; as well as good governance.^61^ However, the GNHI faces some criticism for not addressing human rights principles essential for health.^62^ In the same way that the New Zealand well-being budget^63^ demands that all governmental work is assessed by how it contributes to well-being, Bhutan’s GNHI creates a policy lever based on an alternative conception of the end points of development.^64^

###  Enablers

 Policy levers do not operate in a vacuum. The literature we analysed lays out the features of these contexts – the conditions within which policies emerge, or the conditions under which they are more likely to emerge, which are referred to as enablers.

####  Enabler 1 - Political Will and Accountability

 The mobilization of ‘political will’ as well as the use of targets and mechanisms for accountability surface continually in the literature.^42^ Documents refer to the need for public and political support to enable regulatory reforms.^8,9,40^ Less consideration is given to what would lead to such will or why such will, has not been forthcoming in health or other sectors. Corporate interference is mentioned as a significant barrier.^55^ Calls are, however, made for research to address barriers to the translation of knowledge into action, such as research addressing lack of political will^65^ or decision-making under uncertainty, such as in cases of non-linear, complex interacting forces.^27^ The centrality of political will to enable the right policy levers to be pulled to enhance regulation, and that such will is often needed simultaneously at all or different levels illustrates the interactive dynamics of the cube as per Figure.

 The literature highlights the adoption of transparent accountability mechanisms to cultivate trust across government and between governments and communities, for example, through parliamentary health committees for intersectoral governance.^24^ A key barrier identified is the asymmetry of power, with a vicious cycle whereby certain political and economic institutions (like unions of informal sector workers or indigenous political formations) face disadvantage and have less often been able to shape the economic rules of the game globally, which could, in turn, reduce their disadvantage.^66^ This applies within countries to populations facing historical disadvantage, which may be less represented in decision-making or accountability processes.^21^

 The literature also proposes accountability mechanisms based on human rights principles with independent monitoring bodies reporting, for example, to global platforms and/or greater use of national commissions on health including specifically for intersectoral action.^7,30,37,49,67^ Yet, despite repeated calls for enhanced and independent monitoring to facilitate accountability, such mechanisms remain rare and underdeveloped. Some call for disaggregated data and continued monitoring of health inequalities to facilitate accountability (eg, to leave no one behind), seeing this as a necessary, but not sufficient step to tracking and ensuring political will and accountability.

####  Enabler 2 - Social Mobilization and Community Action

 Many authors point to the power of civil society and social movements, and the power of collective action and alliances,^8,20,27,68^ where the legacy of popular protest, women’s movements, sexual and reproductive rights movements and the HIV movements loom large. In the context of global NCD targets, more recently, civil society organisations have played a key role in “accelerat(ing) political action” and ensuring accountability.^30^ Others call for citizens to directly lobby politicians for action by promoting or protesting actions by government,^55^ and for citizen involvement to challenge government decisions (eg, through mechanisms such as public protest or appointment of ombudspersons).^69^

 The literature repeatedly emphasizes the need for governments to encourage community engagement and the empowerment of community members.^7,8,20,23,67^ Some documents place emphasis on needed mechanisms to enable community mobilisation, and genuine and meaningful participation of communities^9,55,70^ and the resources that would enable it^31,71^ (eg, the Lancet Syndemic Commission called for US$ 1 billion over 10 years to support civil society).^31^ Additionally, these mechanisms should seek to shift norms and structural drivers to address health inequity.^72,73^ One barrier mentioned with respect to inclusion, recommended ensuring that “the least well off [were] included and the interests of powerful groups [were] contained.”^47^

 Frameworks consider questions of representation and diversity in relevant decision-making processes.^3,24,26,50^ The involvement of people living with specific conditions is encouraged through global cooperation and mobilisation.^30^ The literature focuses on the need for people-centred social action^7,22^ and for increased stakeholder engagement, research co-production and capacity including working with health and other sectors.^27,49,70,74^ It is argued that people-centred programme design and implementation could help reduce hierarchies of knowledge.^75^

 Public education^23,51^ is also identified in multiple documents as a critical enabler, in some cases promulgated under the term health literacy.^42,68,76^ As the 1974 Lalonde report on social determinants lays out, informing the public of structural drivers would not merely help protect their own health through individual level behaviour modifications but more importantly enable people to come together, through local organisations to demand action on the social determinants.^17^

####  Enabler 3 - Generation and Use of Knowledge

 Many documents make the case that to drive progress, there is a need for more, and better quality, evidence.^51^ The literature on the social determinants of health underscores the need to use different evidence types, explore research methodologies to understand structural determinants across settings and from different perspectives, and to link research to integrated action strategies.^36^ Methods advocated include ethnographic,^19^ life course and longitudinal,^65^ systems,^31^ complexity, collaborative research approaches (using ‘process innovations’),^75^ as well as policy and programme evaluations.^22,31,76^ Additionally, proposals are made for integrating gender and socio-economic status.^70,77^ There are also calls for the inclusion of perspectives of those (disproportionately and negatively) affected,^28,78,79^ and in particular, to include indigenous and traditional approaches in the knowledge ecosystem.^31^ Calls have been made, and methodologies developed, to share and co-produce knowledge through citizen science methodologies,^51^ and to close the gap between research and practice.

 Given the necessary blurring of boundaries between research, policy, and practice, the importance of individual or organisational agents that explicitly and transparently straddle boundaries, political entrepreneurs and change agents were also found to be crucial for progress.^69^ There are also outstanding methodological challenges; we are at early stages of developing appropriate methods and mechanisms to obtain sufficient evidence on the exact relationship between social determinants and health in specific, actionable contexts,^80^ and much more thought needs to be given to process evaluation associated multisectoral action, policy and governance – for example in relation to civic engagement strategies, prioritised joint action plans and divisions of labour among ministries.

 The reviewed literature stresses the importance of (researchers) carrying out policy research – to understand and address the policy inertia preventing adoption or implementation of progressive, equity-oriented measures.^31^ Whitehead and Dahlgren^70^ for example, argue that a deeper analysis of the “ways in which health systems can confront them [structural determinants] in different contexts” would aid policy-makers. More recently Gilson et al refer to similar processes as “collective sensemaking for action.”^81^ Such approaches are especially critical given the many barriers that exist to leveraging available evidence – particularly that which challenges the status quo. An analysis of the use of evidence during the COVID-19 pandemic calls for attention to governance of evidence itself, by way of scientific advisory systems in government decision-making.^3^ The EAT Forum for global food system transformation was designed as a platform for collaboration to co-create policy-relevant empirical evidence and corresponding solutions,^32^ with some success, but also some important critiques, including its purported endorsement of a “one-size-fits-all” approach.^82^

 The gap between the knowledge of disease and knowledge for implementation, is described as “unacceptable,” especially in LMICs – and the same is true of the creation of health.^27^ The need to fill the gap between scientific evidence and policy making is repeatedly made.^32^ There is some discussion in the literature on building partnerships outside the ministry of health, for example to conduct health impact assessments.^83^ One approach to building partnerships involves learning-by-doing,^1^ where competencies are acquired in a context, directly confronting complexity through application rather than abstract or conceptually focused training. The literature further emphasizes the importance of methodologies relevant to action or the more contextually nuanced approach of realist synthesis.^39,84^ Other documents advocate for “prospective action-oriented applied research.”^27,76^

 Multiple calls are made to generate evidence around “why” and “how” questions and for a shift from focus on individual behaviour to systems: for instance why individuals and communities adopt risky “lifestyle” behaviours and/or consume unhealthy commodities, including tobacco,^40^ alcohol^50^ and unhealthy foods.^76^ The literature also calls for inquiry into “how” structural or upstream drivers of “lifestyle” and “unhealthy” behaviours may be addressed.^31,85^ A political economy lens could identify interests and actors and their power include to frame and normalise the focus on the individual; it could also explore to what extent most individuals have the agency and opportunities to pursue healthy lifestyles. This kind of knowledge is not prominent in this literature, nor is it mainstreamed across health sector thinking and action.

 In some cases, enablers and barriers are shared across multiple health challenges and may drive a synergistic action agenda.^3^ For example, linking obesity with undernutrition and climate change into a single syndemic framework focuses attention on the scale and urgency of addressing these combined challenges and emphasises the need for and benefits of common solutions.^31^

## Discussion

 The selection of texts was made on the basis of frameworks identified in previous reviews; we extended our sample beyond those reviews both qualitatively and quantitatively—several points emerge as discussed below.

###  Where Is the Consideration of Politics and Power?

 The literature has tended to focus on technical, individual-oriented solutions rather than political reform or political-economy informed upstream change. It neglects engagement with civic groups and social movements in political processes.^8^ Of the 27 frameworks looking at the drivers of health equity reviewed by Givens et al, only five consider political or institutional power.^86^ As a result, with a few exceptions,^87^ questions of how to motivate decision makers, how to rally movements, how to engage communities, and how to counteract vested interests are under-articulated and/or unanswered.

 The literature typically does not engage in analysis of power and political-economy; it fails to consider how agendas are advanced and implemented. There were exceptions with some authors arguing that efforts “must be driven by politics.”^69^ The lack of prominence given to politics reflects a positivist, often reductionist and technocratic emphasis of the prevailing and dominant biomedical approach. Useful questions would be around how to disrupt the power of those interests who set the agenda and keep the kind of levers and enablers off such agendas, how to get the public and public health communities to focus on what really matters, how better to frame problems and solutions that mobilize the kind of action called for across frameworks. One example of this could be to establish frameworks focused on societal equity – where health would be one among many sectors where we would seek to remove inequalities and redress the underlying power imbalances that perpetuate them.

 Indeed, one assumption with a lot of intersectoral action models is that decision makers outside the health sector are looking for the health sector to lead action on the social determinants of health. While this may have major implications for action on the structural drivers of health, the health ministry is often unable or unwilling to convene or coordinate other ministries. Moreover, there is a need to question the feasibility of this by policy-makers who tend to privilege bio-medical, technocratic interventions. The type of broad based — equity focused — platform that operates well beyond the paradigms of (only) the health sector, as aforementioned, may offer more strategic and impactful avenues for healthy societies.

 From the perspective of Lukes’ three faces of power heuristic,^88^ the literature does engage with the politics of agenda setting (Lukes’ first face) and to some extent, albeit more limited, on keeping regulatory and fiscal measures off the health agenda (second face). But there is little engagement with the third face of power; the one that perpetuates the status quo in hidden and often unconscious ways – perpetuating power asymmetries or getting people to want things inimical to their interests (such as the over consumption of ultra-processed foods or lower corporate taxes). With an ever-increased concentration of power and wealth in health harming industries, the tools of such power exercised through marketing, promotions and control of print and social media create and shape societal preferences. There is inadequate exploration of the ways in which vested interests and institutions ‘naturalise’ the focus on disease and sickness, diverting attention from a focus on well-being, let alone the development of pro-health, counter-narratives.^89^

 The healthy societies agenda requires an activist or at least progressive interventionist and redistributive government. Yet few of the documents in the sample reflect on the likely or real impact of the structural, institutional and systemic factors introduced or reinforced by neo-liberalism. Few engaged in thinking about how to challenge the prevailing paradigm, where the state has retrenched and the influence of corporate players is substantial.^26,87,90,91^ The period reflected in this paper, when one might have hoped to see the vision and measures outlined in Alma Ata Declaration implemented, was marked at least from the 1980s by a swing away from the state. It was a period during which the post-war social contract in Europe and North America gave way to neo-liberalism. This doctrine was firmly at odds with a large and social-welfare oriented state pulling the above-mentioned levers and investing in the enablers. Trends in many parts of the world represents a move towards authoritarianism and populism, the implications of which are inadequately explored, or ignored altogether.^92,93^

 Community-based partnerships and collaborations between professionals and communities are seen to start to redress imbalances of power, and calls have been made to evaluate such efforts.^51-53,67^ Documents focus on the need for people-centred social action^49,70^ and the need for wider stakeholder engagement including working with health and other sectors.^27,74^ Yet it is only recently that guidelines to support such efforts are emerging.^94^

###  Documentation and Evaluation of What Works

 The literature expands considerably on the nature of the challenges facing healthy societies (the problem space), and yet is thin on assessment of what has worked or not worked to build or move towards them. Evaluations or documentation of successful or failed examples of action are limited. For example, the calls for multi-sector coordination are plentiful, but there is little guidance on what works under what circumstances. There are exceptions, for example, a UNDP report draws on examples of financial and monetary policies to manage climate risks.The WHO Health 2020 framework provides assessments and detailed suggestions, for example, on how to make multi-sectoral action happen — and there are some examples beyond our sample (for example on HiAPs^69^). The extent to which, and importantly why or why not — and for whom, under what circumstances — the levers and enablers described have worked to bring about transformative change would be something to be encouraged to advance the agenda.

 Just as co-benefits can occur from taking integrated approaches to improving health, unintended negative or inequitable impacts can result from well-meaning interventions — both need to be carefully documented.^40,95^ Indeed, particularly if we use a broader societal frame, understanding shared drivers and arriving at co-benefit type approaches will require complexity-based approaches and systems thinking. The methodological challenges in describing and theorizing co-benefits and intersectoral, whole-of-society type approaches, may require a fundamental shift away from or significant adaptations to the dominant modes of measurement and computation that privilege individual level quantitative data.

###  Paradigm Shifts, Mindsets for Change

 Efforts to cast societal well-being in a broader context have been made for the past few decades. The 1990 Human Development Report sought to create an alternative to the gross domestic product, instead “ranking all countries by whether people had the freedom and opportunity to live a life they valued. In so doing, we [UNDP] gave voice to a new conversation on the meaning of a good life and the ways we could achieve it.” Overall, we found that while some of the literature raised this notion of what a good life could be, there was less agreement or even articulation about how this could be achieved.

 While a stream of thinking calls for a redefinition of what constitutes societal progress, our documents did not reflect literature advocating for alternative models of progress to stay within “planetary boundaries.”^96^ Raworth,^97^ O’Neill et al,^98^ Hickel,^99^ and others have laid out social and biophysical threshold levels whereby nations may stay within planetary boundaries — something that is by default achieved in several “poorer” nations and achievable by other global South economies.^100,101^ Concern has been raised that rather than a framing of high-income country or LMIC as monolithic, we must consider the impact of elite population subgroups within high-income country and LMIC contexts, whose negative contribution to planetary health far outstrips that of populations facing disadvantage in either setting.^100,101^

 There was some limited discussion that societal drivers are supra-territorial, needing to be governed at regional and global levels. For example, the Lancet Commission on Planetary Health (2015) looked at the role of regional trade treaties “to further incorporate the protection of health in the near and long term.” It is hard to envision sustained progress on ideas like a circular or well-being economies without broader, paradigmatic shifts. Relatedly, some authors highlight how most planetary health studies were conducted in the global North, and how action and research agendas shaped by such experiences are too narrow.

 There is a need to understand how individual and social power can be organized to advance knowledge, and how this power can be used. In harnessing that power, it will be important that gender, class, disability and other inequities are not further entrenched. This means positively framing healthy and sustainable options for decision-makers and holding governments accountable for the collective right to health and to healthy environments to live, grow, move, work and play.^102^

 The field may, however, need to reconsider accountability. What we found in the literature were general principles on the one hand (eg, calls for transparency) and, on the other, relatively narrow measures. The latter included, for example, undertaking reviews of the practice of intersectoral action for health, establishing independent monitoring boards for specific issues/initiatives or ensuring a forum for monitoring progress on HiAP.^103,104^ Smaller measures, such as investment in the social determinants of health,^102^ could be more effective as part of a wider and systematic effort to hold societies to account for the structural and systemic factors.

 Such reframing has at least three further implications.First, that efforts must be made to identify leadership for this agenda beyond ministries of health. Second, given that the agenda is already large and distributed, there is a need for local priority setting. Third, there is a need for more agile and appropriate methodological approaches, such as systems thinking and political-economy analysis, to guide action.

###  Limitations

 The aims of this work required an expansive literature search; apart from the 68 documents in our sample, we draw upon additional analyses. This zooming in and out of the literature further expanded the literature, and therefore did not meet the criteria of a systematic, close-ended search. As a team of researchers from countries in the Global North and South, our process was iterative and recursive, but constrained by our own positionality. All authors are social scientists with strong orientations towards equity, human rights and social justice. In addition, we were not appraising the validity, reliability or quality of concepts proposed in frameworks or literature we analysed, which would perhaps calibrate or change the emphasis on the various policy levers proposed as well as enablers that emerged. Relatedly, we have crafted our policy levers and enablers to encompass common themes across the literature. Unique themes or other themes with nuances outside of our cube could also emerge from another round of analysis of our data (See Supplementary file 2), but was not included here. Indeed, there may be regional, geographic or other variations that could be looked at in further research on healthy societies, following from Loewenson et al.^105^

 Our focus, on the “how” was not informed by a detailed analysis of barriers to policy action, though we concede that this could have revealed as much as our identification of enablers did. This is a critical area going forward, as indeed, barriers could forestall action on policy levers or otherwise create contexts where even enablers may be vitiated. The analysis was limited to English-language documents, which may be part of why we have a preponderance of documents from high-income, anglophone countries, which creates a bias in terms of perspectives represented in our analysis. Further, our thematic approach has resulted in high-level descriptions with limited quantitative analysis. Finally, it was beyond our purview to map gaps in the research agendas identified as our narrower focus was on the policy and action elements, eg, the “how” of healthy societies. There is a clear role of research in this and of identifying gaps in how research for healthy societies has been framed. Identifying and then filling these research gaps is another area of future work in this space.

## Conclusion

 This paper has explored the “how” of healthy societies, finding some recurring themes, ideas and approaches promoted in the literature to achieve them. The literature identifies policy levers and enablers, but does not provide a closer diagnosis of why such levers have not been leveraged. Despite continuous advocacy around generating political will, many documents continued to view levers as technical instruments. Yet levers are connected to political-economic arrangements associated with the prevailing socio-economic paradigms as are decisions to invest (or not) in enablers. Apolitical approaches do not create healthy societies. This represents a failure of our collective imagination and a major missed opportunity to those who seek Health for All. By ceding space for more progressive sources of power to emerge, the paralyzing limits of the healthcare system may be transcended, and our minds and societies opened to more radical possibilities for a future focused on systems for health and the ensuing health and well-being.^106^

## Acknowledgements

 The authors would like to acknowledge Sreejini Jaya for initial data extraction and coding.

## Ethical issues

 Not applicable.

## Competing interests

 Robert Marten and Sonam Yangchen are staff members of the Alliance for Health Policy and Systems Research, a WHO-hosted partnership.

## Funding

 This research received funding from The Alliance for Health Policy and Systems Research.

## Endnotes

 [1] As described in our companion paper, we carried out Google Scholar searches (using terms ‘Healthy Societies + Framework,’ ‘Health + Framework,’ ‘Health + Societies,’ ‘Healthy Societies + approach,’ ‘Health Systems + Framework’ covering period 1974 to 2022), yielding 31 included documents. Another 25 documents were included proposed by the senior authors as relevant to the study aims or through snowballing.

## Supplementary files

Supplementary file 1. Preferred Reporting Items for Systematic Reviews and Meta-Analyses Extension for Scoping Reviews (PRISMA-ScR) Flow Diagram.

Supplementary file 2. Documents Included in the Review.
