# Supplementary material for: How to Build Healthy Societies: A Thematic Analysis of Relevant Conceptual Frameworks
Source: Int J Health Policy Manag. 2023 Nov 7;12:7451. doi: 10.34172/ijhpm.2023.7451 (PMC10699821; doi:10.34172/ijhpm.2023.7451)
Supplement: Supplementary file 1 — Preferred Reporting Items for Systematic Reviews and Meta-Analyses Extension for Scoping Reviews (PRISMA-ScR) Flow Diagram. [file ijhpm-12-7451-s001.pdf]

Article title: How to Build Healthy Societies: A Thematic Analysis of Relevant Conceptual Frameworks

Journal name: International Journal of Health Policy and Management (IJHPM)

Authors' information: Devaki Nambiar[1,2,3], Amy Bestman[2], Siddharth Srivastava[1], Robert Marten[4], Sonam Yangchen[4], Kent Buse[5]*

[1]The George Institute for Global Health, New Delhi, India.

[2]Faculty of Medicine, University of New South Wales, Sydney, NSW, Australia.

[3]Prasanna School of Public Health, Manipal Academy of Higher Education, Manipal, India.

[4]The Alliance for Health Policy and Systems Research, World Health Organization (WHO), Geneva, Switzerland.

[5]The George Institute for Global Health, Imperial College London, London, UK.

***Correspondence to:** Kent Buse; Email: kentbuse@gmail.com

Citation: Nambiar D, Bestman A, Srivastava S, Marten R, Yangchen S, Buse K. How to build healthy societies: a thematic analysis of relevant conceptual frameworks. Int J Health Policy Manag. 2023;12:7451. doi:10.34172/ijhpm.2023.7451

Supplementary file 1. Preferred Reporting Items for Systematic Reviews and Meta-Analyses Extension for Scoping Reviews (PRISMA-ScR) Flow Diagram

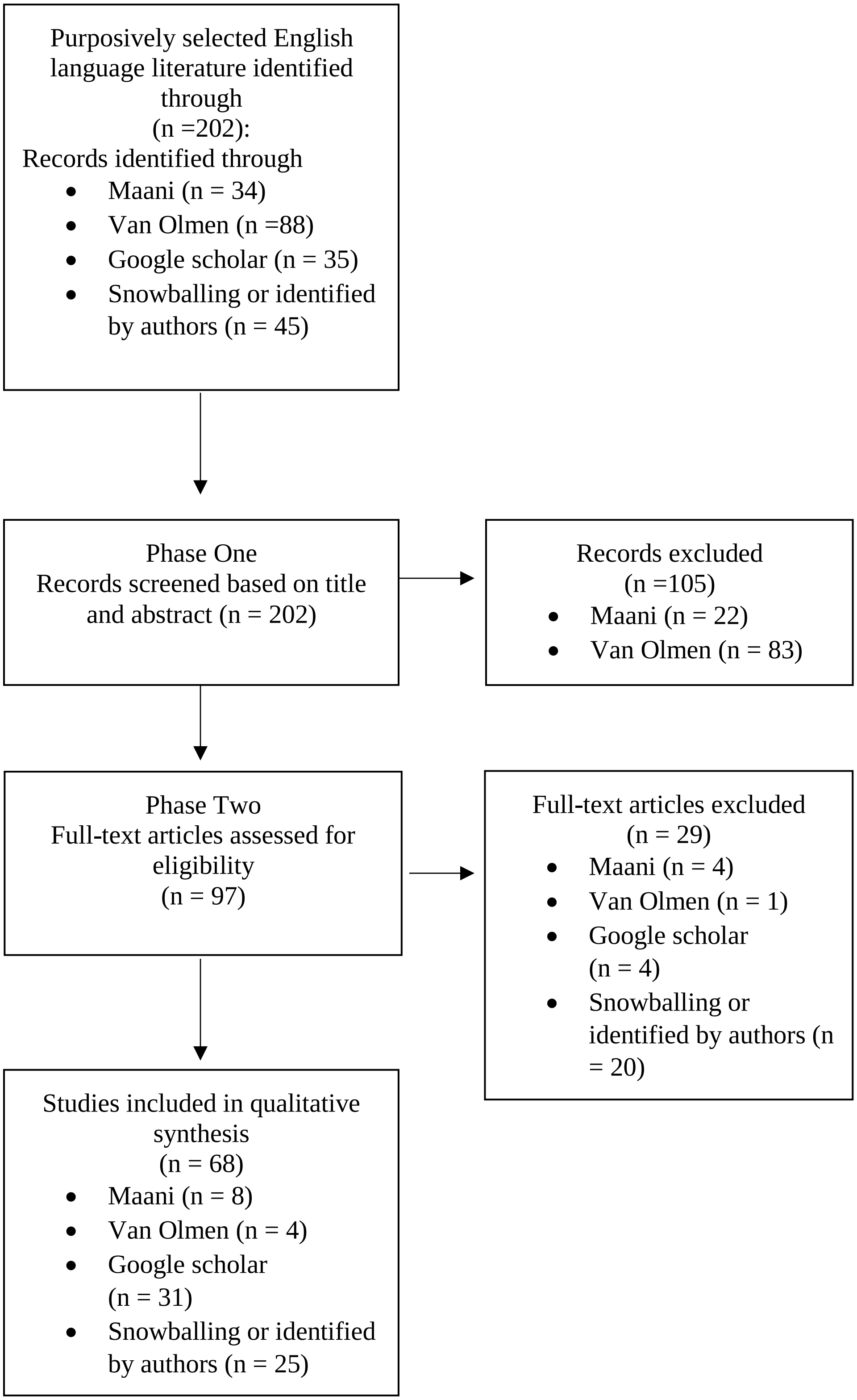
Purposively selected English language literature identified through (n =202):
Records identified through
Maani (n = 34)
Van Olmen (n =88)
Google scholar (n = 35)
Snowballing or identified by authors (n = 45)
Phase One
Records screened based on title and abstract (n = 202)
Records excluded (n =105)
Maani (n = 22)
Van Olmen (n = 83)
Phase Two
Full-text articles assessed for eligibility (n = 97)
Full-text articles excluded (n = 29)
Maani (n = 4)
Van Olmen (n = 1)
Google scholar (n = 4)
Snowballing or identified by authors (n = 20)
Studies included in qualitative synthesis (n = 68)
Maani (n = 8)
Van Olmen (n = 4)
Google scholar (n = 31)
Snowballing or identified by authors (n = 25)
