# Supplementary material for: How to Build Healthy Societies: A Thematic Analysis of Relevant Conceptual Frameworks
Source: Int J Health Policy Manag. 2023 Nov 7;12:7451. doi: 10.34172/ijhpm.2023.7451 (PMC10699821; doi:10.34172/ijhpm.2023.7451)
Supplement: Supplementary file 2 — Documents Included in the Review. [file ijhpm-12-7451-s002.pdf]

Article title: How to Build Healthy Societies: A Thematic Analysis of Relevant Conceptual Frameworks

Journal name: International Journal of Health Policy and Management (IJHPM)

Authors' information: Devaki Nambiar[1,2,3], Amy Bestman[2], Siddharth Srivastava[1], Robert Marten[4], Sonam Yangchen[4], Kent Buse[5]*

[1]The George Institute for Global Health, New Delhi, India.

[2]Faculty of Medicine, University of New South Wales, Sydney, NSW, Australia.

[3]Prasanna School of Public Health, Manipal Academy of Higher Education, Manipal, India.

[4]The Alliance for Health Policy and Systems Research, World Health Organization (WHO), Geneva, Switzerland.

[5]The George Institute for Global Health, Imperial College London, London, UK.

***Correspondence to:** Kent Buse; Email: kentbuse@gmail.com

Citation: Nambiar D, Bestman A, Srivastava S, Marten R, Yangchen S, Buse K. How to build healthy societies: a thematic analysis of relevant conceptual frameworks. Int J Health Policy Manag. 2023;12:7451. doi:10.34172/ijhpm.2023.7451

Supplementary file 2. Documents Included in the Review

Year	Author	Framework/ document title	Type of document	Levers & enablers
**1974**	Lalonde[17]	A new perspective on the health of Canadians: A working document.	Working document	RFM<br>IA<br>RP
**1978**	WHO[7]	Primary health care: report of the International Conference on primary health care, Alma-Ata	Conference declaration	RFM<br>PWA<br>SM
**1986**	WHO Euro[8]	Ottawa Charter for Health Promotion	Conference charter	RFM<br>IA<br>RP<br>PWA<br>SM
**1993**	Sen[107]	Capability and Well-Being	Book chapter	SM
**1996**	Hamilton & Bhatti[108]	Population health promotion: An integrated model of population health and health promotion.	Government working document	GK<br>RS
**2000**	Berkman et al.[109]	From social integration to health: Durkheim in the new millennium	Review	GK
**2000**	People 's Health Movement[29]	The People's Charter for Health	Conference charter	RFM<br>IA<br>PWA<br>SM<br>KG
**2001**	Participants of the conference on "Health Care for All"[37]	Declaration on 'health care for all'	Conference declaration	RFM<br>IA<br>PWA<br>GK
**2003**	Chen & Narasimhan[49]	Human security and global health	Journal article	PWA<br>RP
**2003**	Ogata & Sen[110]	Human Security Now: Commission on Human Security	Commission Report	RFM<br>IA<br>RP

Year	Author	Framework/ document title	Type of document	Levers & enablers
				SM<br>GK
**2004**	Schulz & Northridge[83]	Social determinants of health: implications for environmental health promotion	Journal article	IA<br>RP<br>PWA
**2005**	Gasper	Securing humanity: situating ‘human security’ as concept and discourse	Journal article	PWA
**2006**	Barton & Grant[38]	A health map for the local human habitat	Editorial	IA
**2006**	Dahlgren & Whitehead[19]	European strategies for tackling social inequities in health: Levelling up Part 2	WHO report	RFM<br>IA<br>RP<br>SM<br>GK
**2006**	Etches et al.[47]	Measuring population health: a review of indicators	Journal article	RP<br>GK
**2006**	Ståhl et al.[23]	Health in all policies: prospects and potentials	Project report	RFM<br>IA<br>PWA<br>SM<br>GK
**2006**	Whitehead & Dahlgren[70]	Concepts and principles for tackling social inequities in health: Levelling up Part 1	WHO report	PWA<br>SM<br>GK
**2007**	Siddiq et al.[22]	Total environment assessment model for early child development: evidence report for the World Health Organization’s Commission on the social determinants of health	Evidence report	RFM<br>IA<br>SM<br>GK
**2007**	Whitehead[84]	A typology of actions to tackle social inequalities in health.	Editorial	IA<br>GK
**2008**	Commission on Social Determinants of Health.[9]	Closing the gap in a generation: health equity through action on the social determinants of health: final report of the commission on social determinants of health	Comission report	RFM<br>IA<br>RP<br>PWA<br>SM<br>GK
**2008**	Dyck[78]	Social determinants of Métis health. Canada: Métis Centre	Report	RP<br>SM<br>GK
**2008**	Hiatt & Breen[111]	The social determinants of cancer: a challenge for transdisciplinary science	Journal article	IA<br>GK
**2008**	Kawachi et al.[71]	Social Capital and Health: A Decade of Progress and Beyond	Book chapter	SM<br>GK
**2009**	Fox & Meier[66]	Health as freedom: addressing social determinants of global health inequities through the human right to development	Journal article	RFM<br>PWA
**2010**	Bambra et al.[112]	Tackling the wider social determinants of health and health inequalities: evidence from systematic reviews	Journal article	IA<br>GK
**2010**	Bozorgmehr[113]	Rethinking the 'global' in global health: a dialectic approach	Journal article	RFM<br>PWA<br>GK
**2010**	Solar & Irwin[20]	A conceptual framework for action on the social determinants of health	WHO discussion paper	RFM<br>IA<br>RP<br>PWA

Year	Author	Framework/ document title	Type of document	Levers & enablers
				SM<br>GK
**2011**	Braveman et al.[65]	The social determinants of health: coming of age	Journal article	IA<br>PWA<br>SM
**2012**	Golden & Earp[114]	Social ecological approaches to individuals and their contexts: twenty years of health education & behavior health promotion interventions	Journal article	GK
**2012**	Lorenc et al.[115]	Crime, fear of crime, environment, and mental health and wellbeing: mapping review of theories and causal pathways	Journal article	GK
**2013**	Board on Population Health Public Health Practice et al.[52]	US health in international perspective: Shorter lives, poorer health	Book	RP<br>SM<br>GK
**2013**	WHO Regional Committee for Europe[24]	Health 2020: a European policy framework and strategy for the 21st century	WHO policy framework	RFM<br>IA<br>RP<br>PWA<br>SM<br>GK
**2014**	Krumeich & Meershoek[80]	Health in global context; beyond the social determinants of health?	Journal article	GK
**2014**	Welsh et al.[53]	Evidence Review: Addressing the social determinants of inequities in mental wellbeing of children and adolescents	Evidence review	IA<br>RP<br>GK
**2014**	WHO[42]	Health in all policies: Helsinki statement. Framework for country action.	Conference statement and framework	RFM<br>IA<br>PWA<br>SM<br>GK
**2015**	Ball et al.[95]	Evidence review: addressing the social determinants of inequities in physical activity and related health outcomes	Evidence review	RFM<br>IA<br>GK
**2015**	Friel et al.[76]	Evidence review: Addressing the social determinants of inequities in healthy eating	Evidence review	RFM<br>IA<br>PWA<br>GK
**2015**	Newman et al.[35]	Evidence Review: Settings for Addressing the Social Determinants of Health Inequities	Evidence review	RFM<br>IA<br>SM<br>GK
**2015**	Purcell [40]	Evidence review: Addressing the social determinants of inequities in tobacco use	Evidence review	RFM<br>IA<br>PWA<br>GK
**2015**	VicHealth.[116]	Evidence review: the social determinants of inequities in alcohol consumption and alcohol-related health outcomes	Evidence review	RFM<br>RS<br>PWA<br>SM<br>GK
**2015**	VicHealth[117]	Fair Foundations: the VicHealth Framework for Health Equity	Conceptual framework	RFM<br>IA<br>PWA<br>SM<br>GK
**2015**	VicHealth[67]	Promoting equity in early childhood development for health equity through the life course	Evidence review	RFM<br>IA<br>SM<br>GK

Year	Author	Framework/ document title	Type of document	Levers & enablers
**2015**	VicHealth[75]	Promoting equity through social innovation	Evidence review	IA<br>SM<br>GK
**2015**	Whitmee et al.[27]	Safeguarding human health in the Anthropocene epoch: report of The Rockefeller Foundation–Lancet Commission on planetary health.	Commission report	RFM<br>IA<br>RP<br>PWA<br>SM<br>GK
**2016**	Attendees of the 9th Global Conference on Health Promotion[68]	Shanghai Declaration on promoting health in the 2030 Agenda for Sustainable Development	Conference declaration	RFM<br>IA<br>SM
**2016**	Graham & White[39]	Social determinants and lifestyles: integrating environmental and public health perspectives	Journal article	IA<br>SM<br>GK
**2017**	Buse et al.[26]	Healthy people and healthy profits? Elaborating a conceptual framework for governing the commercial determinants of non-communicable diseases and identifying options for reducing risk exposure.	Journal article	RFM<br>IA<br>PWA<br>SM
**2017**	Boswell et al.[74]	Keeping Us Well: How Non-health Charities Address the Social Determinants of Health	Report	IA
**2017**	de Leeuw[69]	Engagement of Sectors Other than Health in Integrated Health Governance, Policy, and Action	Journal article	RFM<br>IA<br>SM<br>GK
**2017**	McNamara[28]	Trade liberalization and social determinants of health: a state of the literature review	Journal article	RFM<br>GK
**2018**	Koehler et al.[51]	Building healthy community environments: a public health approach	Journal article	IA<br>GK<br>RP
**2018**	Kondo et al.[79]	Urban green space and its impact on human health	Journal article	GK
**2018**	WHO Independent High-level Commission on NCDs[118]	Report of the Technical Consultation (21-22 March 2018)	WHO report	RFM<br>IA<br>PWA<br>SM<br>GK
**2018**	Vik & Carlquist[54]	Measuring subjective well-being for policy purposes: The example of well-being indicators in the WHO "Health 2020" framework	Journal article	GK<br>RP
**2019**	Cerf[119]	Sustainable Development Goal Integration, Interdependence, and Implementation: the Environment–Economic–Health Nexus and Universal Health Coverage	Journal article	IA<br>PWA
**2019**	Government of New Zealand[63]	The Wellbeing Budget	Government document	RFM<br>IA<br>RP<br>PWA<br>SM
**2019**	Swinburn et al.[31]	The global Syndemic of obesity, undernutrition, and climate change: the Lancet Commission report	Commission report	RFM<br>IA<br>PWA<br>SM<br>GK

Year	Author	Framework/ document title	Type of document	Levers & enablers
**2019**	Verma[59]	The Eight Manifestations of GNH: Multiple Meanings of a Development Alternative	Journal article	RP
**2019**	WHO[14]	Thirteenth General Programme of Work 2019–2023	General program of work	RFM IA RP RFM SM GK
**2019**	Willett et al.[32]	Food in the Anthropocene: the EAT–Lancet Commission on healthy diets from sustainable food systems	Lancet Commission report	RFM IA SM GK
**2020**	Amuasi et al.[36]	Reconnecting for our future: the Lancet One Health commission	Commission report	IA GK
**2020**	Hawkes & Buse[120]	The Politics of Gender and Global Health	Book chapter	PWA SM GK
**2020**	Herrick & Bell[77]	Concepts, disciplines and politics: on 'structural violence' and the 'social determinants of health	Journal article	N/A
**2020**	Raphael et al.[55]	Social Determinants of Health: The Canadian Facts, 2nd Edition	Book	RFM IA RP SM GK
**2020**	United Nations [3]	UN Research Roadmap for the COVID-19 Recovery: Leveraging the Power of Science for a More Equitable, Resilient and Sustainable Future	Research roadmap	RFM IA PWA SM GK
**2020**	United Nations Development Programme[1]	Human Development Report 2020: The next frontier Human development and the Anthropocene	Report	RFM IA RP PWA SM GK
**2021**	Lacy-Nichols & Marten[87]	Power and the commercial determinants of health: ideas for a research agenda	Journal article	RFM
**2021**	US House of Representatives [21]	A Bold Vision for a Legislative Path Toward Health and Economic Equity	Committee Report	RFM IA RP SM

Levers and enablers RFM-Regulatory and fiscal measures; IA- Intersectoral action, RP- Redefining measures of progress, PWA-Political will and accountability, SM- Social mobilization and community action; GK- Generation and use of knowledge
